# P04 Solution fundamentals are lacking: time to seek practical ways to tackle the AMR crisis in Sudan and other similar settings

**DOI:** 10.1093/jacamr/dlac053.004

**Published:** 2022-05-31

**Authors:** Rehab Ahmed

**Affiliations:** 1 Faculty of Pharmacy, University of Tabuk, Tabuk, Saudi Arabia; 2 Faculty of Pharmacy, University of Khartoum, Khartoum, Sudan

## Abstract

**Background:**

A flood of data is showing that the silent pandemic is no longer silent. It is already here and impacting the entire world and is particularly severe in low- and middle-income countries.

**Objectives:**

To highlight the scarcity of data about antimicrobial resistance (AMR) in Sudan, which is now devastated by the political unrest. Simultaneously the review is calling attention to the day-to-day deterioration in the infrastructural foundation necessary to combat AMR.

**Methods:**

The review spans different topics related to AMR. It includes data and epidemiology of antimicrobial resistance, awareness and education about AMR, awareness about immunization, healthcare infrastructure, pharmacists’ viewpoints of their current roles in the healthcare.

**Results:**

Few data about AMR are published. Resistance is reported mostly based on phenotypic data. Huge gaps in the knowledge, awareness and the curricula are evidenced to be present even among medical and veterinary students. Inefficiency of regulatory bodies and lack of coordination between them is reported. Published data reflect a wide acceptance of immunization in children under 5 years old. Pharmacists are unsatisfied by their current jobs and are ready to participate better in healthcare.

**Conclusions:**

The radical solutions to AMR entail building robust infrastructure for healthcare and acquiring political will, which are currently and in the foreseeable future impractical and unattainable. However, solution opportunities still exist. I see three main opportunities represented in pharmacists, immunization programmes and non-profit organizations, mainly WHO. A great deal of potential lies in the sector of pharmacists. There is a substantial number of them who are willing to contribute better to healthcare. One example is the effective engagement of pharmacists who are graduating in high qualifications and education while doing minor roles. Their engagement could be incentivized by considering the participation part of their professional growth. Contrary to the case of adults, immunization of children under 5 years is accepted and implemented largely in Sudan even in remote areas. This is the second potential solution that is worthy of investment and would pay off. WHO and similar organizations at the very least could play the role of the missing coordinator, or even better they could devise pioneering know-how programmes ready to be implemented in these countries. In these programmes, WHO will be able to attract a lot of qualified volunteers to participate in awareness and education campaigns. They also could engage higher education institutions and ministries of health.

